# Silver nanoparticles synthesized by probiotic bacteria and antibacterial role in resistant bacteria

**DOI:** 10.1186/s13568-023-01651-7

**Published:** 2023-12-07

**Authors:** Eman Khalifa, Mohamed Abdel Rafea, Nazir Mustapha, Rania Sultan, ElSayed Hafez

**Affiliations:** 1 Department of Microbiology, Faculty of Veterinary Medicine, Matrouh University, Matrouh, 51511 Egypt; 2 Department of Physics, College of Science, Imam Mohammad Ibn Saud Islamic University (IMSIU), Riyadh, 11623 Kingdom of Saudi Arabia; 3 Department of Biological Sciences, Faculty of Science, King Abdulaziz University, Jeddah, 21589 Saudi Arabia; 4 Department of Plant Protection and Biomolecular Diagnosis, ALCRI, City of Scientific Research and Technological Applications Alexandria, Alexandria, Egypt

**Keywords:** *Lactobacillus*, Nanosilver, Cytotoxicity, Antibacterial, Sequencing, AgNPS, Probiotic, MDR

## Abstract

Many dangerous bacteria have become highly resistant to traditional antibiotics, which is a huge public health concern. This study investigated the use of silver nanoparticles biosynthesized in a culture filtrate of *Lactobacillus acidophilus* as antimicrobials. UV–visual spectrophotometry, Fourier-transform-infrared spectroscopy, X-ray power diffraction, and scanning electron microscopy have all validated the findings. The biosynthesized nanoparticles ranged in size from 33 to 90 nm. The cytotoxicity of the nanosilver generated was then investigated using nine 200 g BW rats separated into three groups. When compared to the control group, the treated rats showed little signs of toxicity; parameters of physiological function, including alanine transaminase, aspartate aminotransferase, albumin, creatinine, and urea were significantly different in treated and non-treated animals. Moreover, the antibacterial role of the generated silver nanoparticles was examined in multi-drug resistant (MDR) pathogenic bacteria, *Proteus vulgaris*, *Escherichia coli*, *Staphylococcus aureus*, and *Klebsiella pneumoniae*, revealing high antibacterial activity against the examined bacteria. For more demonstration of the effect of the nanosilver on transcription and gene regulation of treated and non-treated bacteria differential display droplet digital-PCR was used, and the results revealed that several genes were up- and down-regulated. Some genes were selected for DNA sequencing and according to the sequence analysis, these genes were *mec*A, beta-lactam, and unidentified protein genes, and these have been deposited in the GenBank Database with the following accession numbers: *Staphylococcus* MZ748472 and *Klebsiella* MZ748473. We conclude that silver nanoparticles biosynthesized by *L. acidophilus* are environmentally friendly and have antibacterial activities against MDR pathogenic bacteria.

## Key Points

- Our gifted results established that we were right for renewed interest of AgNPs.
- The AgNPS, can be used to prevent treatment failure.
- MDR bacteria are of a great health burden and should be overcomed.

## Introduction

The resistance of pathogenic bacteria to conventional antibiotics is a serious threat to health worldwide. Bacterial resistance to antibiotics is a major therapeutic problem (Levy 1992). Methicillin-resistant *Staphylococcus aureus* (MRSA), vancomycin-resistant enterococci multi-drug-resistant *Mycobacterium tuberculosis* (MDRTB) strains, and multi-drug-resistant (MDR) Gram-negative bacteria are frequently found. Some bacteria are inherently resistant to antibiotics, while others have acquired resistance to antibiotics once widely used to treat them, although no pharmaceutical intervention is devoid of problems associated with resistance (Nikaido 1998). When bacteria multiply in the presence of antibiotics, resistance develops along with a genetic mutation. The mutant gene can easily be passed on to other bacteria (Enright et al. 2002).

Developing a dependable, environmentally acceptable process for nanomaterial synthesis is a difficult task. *L. acidophilus* filtrate has been used as a reduction and capping agent in biosynthesis of silver nanoparticles (AgNPs) that have potential antimicrobial properties against *Klebsiella pneumoniae* (Rajesh et al. 2015). AgNPs thus created were examined in Gram-negative *E. coli* and *Pseudomonas aeruginosa* and it was shown that all antibiotic-resistant bacterial species are highly sensitive to silver nanoparticles biosynthesized by *L. acidophilus* (Cathrine et al. 2010). The development of nanoparticles with a variety of biologic properties is gaining popularity (Abdel-Azeem et al. 2020). Nanomaterials have unique structures with unique physical, chemical, and biological properties; as a result of their nanoscale size, nanomaterials have a wide range of applications (Stoimenov et al. 2002). Nanotechnology may be a useful tool for controlling a wide range of diseases, and this could be accomplished by delivering nanoparticles as functional molecules and/or diagnostic tools for specific diseases. Nanotechnology is also being assessed in agricultural diseases and food safety (Sharon et al. 2010). Antimicrobial nanoparticles could be utilized to fight a variety of infections (Wani et al. 2012). Because of their unusual features, AgNPs apart from their antibacterial and antifungal qualities have received much attention in recent years (Krzyzanowski et al. 2021).

Microorganisms are known to be very susceptible to Ag ions and Ag-based compounds. Bio-AgNPs were found to have significant biocidal effects on as many as 12 bacterial species, including *E. coli* (Chen and Schluesener 2008). Silver has traditionally been used to suppress bacterial growth in a number of settings, such as dental procedures and burn lesions (Catauro et al. 2004; Crabtree et al. 2003). AgNPs have been shown to be an effective biocide against a wide range of bacteria, including Gram-positive and Gram-negative bacteria (Jones and Hoek 2010). Many studies have emphasized AgNPs as a promising strategy for the synthesis of new antibacterial agents (Youssef and Elamawi 2018).

The tests that are performed on the liver; liver function tests; being misleading because many of the tests do not assess the liver’s function but rather identify the source of the damage. Hepatocellular disease is defined by elevated ALT and AST levels that are out of proportion to ALP and bilirubin. The ability of the liver to manufacture albumin and vitamin K-dependent clotting factors can be used to assess its real function (Ribeiro et al. 2019).

β-Lactam antibiotics, for example penicillin and methicillin, are among the most potent and oldest antibiotics that inhibit the formation of bacterial cell walls. Some bacteria produce the lactamase enzyme, which breaks down the β-lactam ring. Bacteria that express the *mec*A gene, which encodes low-binding-affinity penicillin-binding proteins, are another source of resistance; methicillin-resistant *S. aureus* (MRSA) have adapted this method as well (Rice 2012).

## Materials and methods

### Bacterial strains

All the bacterial strains involved in this study are brought from MIRCEN (Ain Shames University, Egypt): *Staphylococcus aureus* EMCC 1351, *Escherichia coli* ATCC 25922, *Klebsiella pneumoniae* EMCC 1637, *Lactobacillus acidophilus* EMCC 1324 and *Proteus vulgaris* ATCC 8427.

### Preparation of *L. acidophilus* filtrate and silver nitrate reduction

The reduction of silver nitrate yielded bionanosilver with *Lactobacillus acidophilus* filtrate according to the method of Rajesh et al. (2015) as Silver nanoparticles were made using deionized water as a solvent. *L. acidophilus* starter culture was incubated in Man-Rogosa-Sharpe broth overnight at 37 °C for 48 h. Following incubation, the culture was centrifuged for 30 min at 10,000 rpm to separate the supernatant using Whatmann filter paper no. 1. After that, different ratios of 2:8, 3:7, and 4:6 of culture filtrate to silver nitrate (Sigma, Aldrich) solution were applied to the culture filtrate. The synthesis was then continued for 24 h at 35 °C in the dark. Silver nanoparticle isolation and purification Centrifugation was used to separate the silver nanoparticles and it took 20 min at 10,000 rpm. Two times, sterile distilled water was used to wash the pelleted silver nanoparticles.

### Spectral study of UV–visible light

A UV–visible spectrophotometer set to 420 nm was used to track the biosynthesis of AgNPs on a regular basis; 0.1 mL of the sample was obtained and diluted to 2 mL with deionized water for the analysis (El-Aassar et al. 2020).

### Pathogenic bacteria

Luria–Bertani (LB) broth media (Becton-Dickinson and Co., USA) were used to culture the bacteria (*P. vulgaris, E. coli*, *S. aureus*, and *K. pneumoniae*). One-mL aliquots of a 24-h-old broth culture of the tested microorganisms were aseptically disseminated onto nutrient agar slopes and incubated for 24 h at 37 °C. The bacterial growth was extracted and washed away with sterile normal saline, yielding a solution with roughly 10^8^–10^9^ CFU/mL. The suspension was kept refrigerated at 4 °C until it was used (Al-Askar et al. 2013).

### Cytoxicological effects of bionanosilver on experimental animals

Nine rats about 200 g in weight were divided into three groups to study nanoparticle toxicity in vivo. The three groups were G1, intragastric application with 200 mg/kg AgNPs, sacrificed after 1 week; G2, animals given the same dose but kept for 2 weeks; and G3, the control animals (Lala et al. 2022).

### Antibacterial activity of AgNPs

The antibacterial efficacy of the generated AgNPs against harmful bacteria was investigated according to the method of Al-Askar et al. (2013). In addition, biogenic antibiotics (200 µg/mL) such as ampicillin, gentamicin, and streptomycin were used to test the nanoparticles’ bactericidal activity.

### Bionanosilver characterization using scanning electron microscopy

One drop of the created AgNPs aqueous suspension was put on a glass slide, and the films on the SEM grids were allowed to dry for 2 min. SEM analysis was carried out on a Hitachi-JP/H7600 apparatus (Japan) with a 100 kV accelerating voltage. With the use of SigmaScan Pro software, the size of the resulting AgNPs was determined based on SEM micrographs (SPSS Inc., Version 4.01.003). Energy dispersive X-ray studies were performed on a JEOL JSM-6400 microscope (Japan) (El-Aassar et al. 2020).

### X-ray diffraction and fourier-transform-infrared spectroscopy

X-ray power diffraction (XRD) (Shimadzu XRD 7000 X-ray diffractometer, Japan) was used to explore the structure of the copolymer as well as the formation of AgNPs, and the structure of the AgNPs was evaluated using Fourier-transform-infrared (FTIR) spectra. To make pellets, powder samples were combined with potassium bromide. A Bruker TENSOR Series FTIR spectrometer (Germany) connected to a PC was used to record FTIR spectra in the absorbance mode, and the data were analyzed using IR Solution OPUS^™^ software (El-Aassar et al. 2020).

### Analyzing particle size

A submicron particle size analyzer was used to examine the particle size of AgNPs during their formation (Beckman Coulter, USA). The sample was dispersed in water at 20 °C with a viscosity of 1.002 and a refractive index of 1.33 (El-Aassar et al. 2020).

### Total RNA extraction and cDNA synthesis from treated and untreated bacteria

An RNeasy Mini Kit was used to isolate bacterial RNA from both treated and untreated bacteria, according to the manufacturer’s recommendations (Qiagen, Germany). The isolated RNA was treated with DNase for 1 h at 37 °C to eliminate any DNA residue. Reverse transcription of purified total RNA into cDNA was performed in a reaction volume of 25 µL. Included in the reaction mixture were 2.5 µL of 5× buffer with MgCl_2_, 2.5 µL of 2.5 mM dNTPs, 4 µL of oligo (dT) primer (20 pml/L), 2 g RNA, and 200 U reverse transcriptase enzyme (MLV, Fermentas, USA). A thermal cycler (Eppendorf, Germany) was used to perform RT-PCR amplification at 42 °C for 1 h and 72 °C for 10 min. The cDNA was then kept at − 20 °C until it was needed (El-Aassar et al. 2020).

### PCR with differential display

The reverse primers of the *mec*A and beta-lactam genes were used to trace the mRNA of the two genes in the bacteria treated with biosynthesized nanosilver. For *mec*A, R: 5′-AGTGCAGTA CCGGATTTGC-3′ (Kumurya 2015). For beta-lactam, R: 5′-GAGCTCGGTATTGTAATATGATCCTCTAGA-3′ (Jiang et al. 2020). A total amount of 25 µL of the droplet-digital PCR (DD-PCR) reaction mixture was used according to Ramadan (2015). The PCR amplification was carried out in an Eppendorf thermal cycler, which was designed for one cycle at 95 °C for 5 min, followed by 34 cycles as follows: Denaturation at 30 s at 95 °C, annealing 1 min at 45–48 °C, and elongation at 1 min at 72 °C. As a last extension, the reaction was incubated at 72 °C for 10 min. Prior to loading 10 µL per gel slot, 2 µL of loading dye was applied. In 1.5% agarose/0.5× TBE gels, electrophoresis was done at 80 v using 0.5× TBE as running buffer, and the gel was subsequently stained in 0.5 µg/mL (w/v) ethidium bromide solutions and destained in deionized water. Finally, a gel documentation system was used to visualize and photograph the gel. The STATISTICA5 tool was used to evaluate the data based on the up-down-regulated genes.

### Sequencing of selected bands and DNA sequence analysis

Four distinct bands (three down-regulated and one uncontrolled) were chosen and purified from the gel using a PCR purification kit (Qiagen, USA), and the purified DNA was sent to be sequenced (Macrogene, Korea). The sequence analysis was performed using the CLUSTALW program (https://www.genome.jp/tools-bin/clustalw) and the phylogeny was constructed using the MEGA 5 program (https://www.megasoftware.net/).

### Analytical statistics

Collected data were processed then reported as mean plus or minus standard deviation (mean ± S.D.). Using Excel, one-way-ANOVA (Microsoft Office 2007) was performed to assess the effect of prepared AgNPs on various physiological parameters. A value of *P* < 0.05 was considered to be statistically significant.

## Results

### Silver nitrate reduction and nanosilver formation

**Fig. 1:**
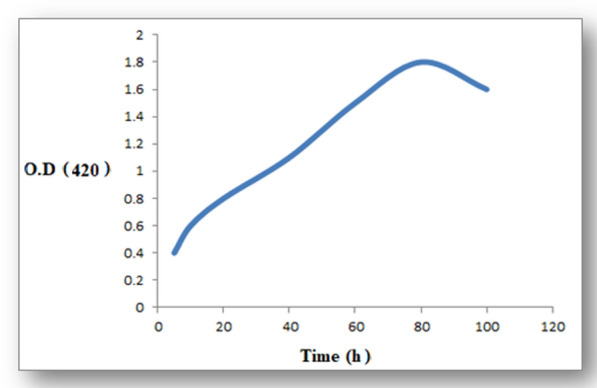
The OD_420_ of the formed AgNPs by *Lactobacillus acidophilus*

The OD 420 increase began in the first 5 h, then reached its peak at 80 h, and then declined after 100 h of incubation (Fig. 1). After 10 days of incubation, the color of *L. acidophilus* filtrate shifted from yellow to dark brown.

### SEM observations of AgNPs

**Fig. 2:**
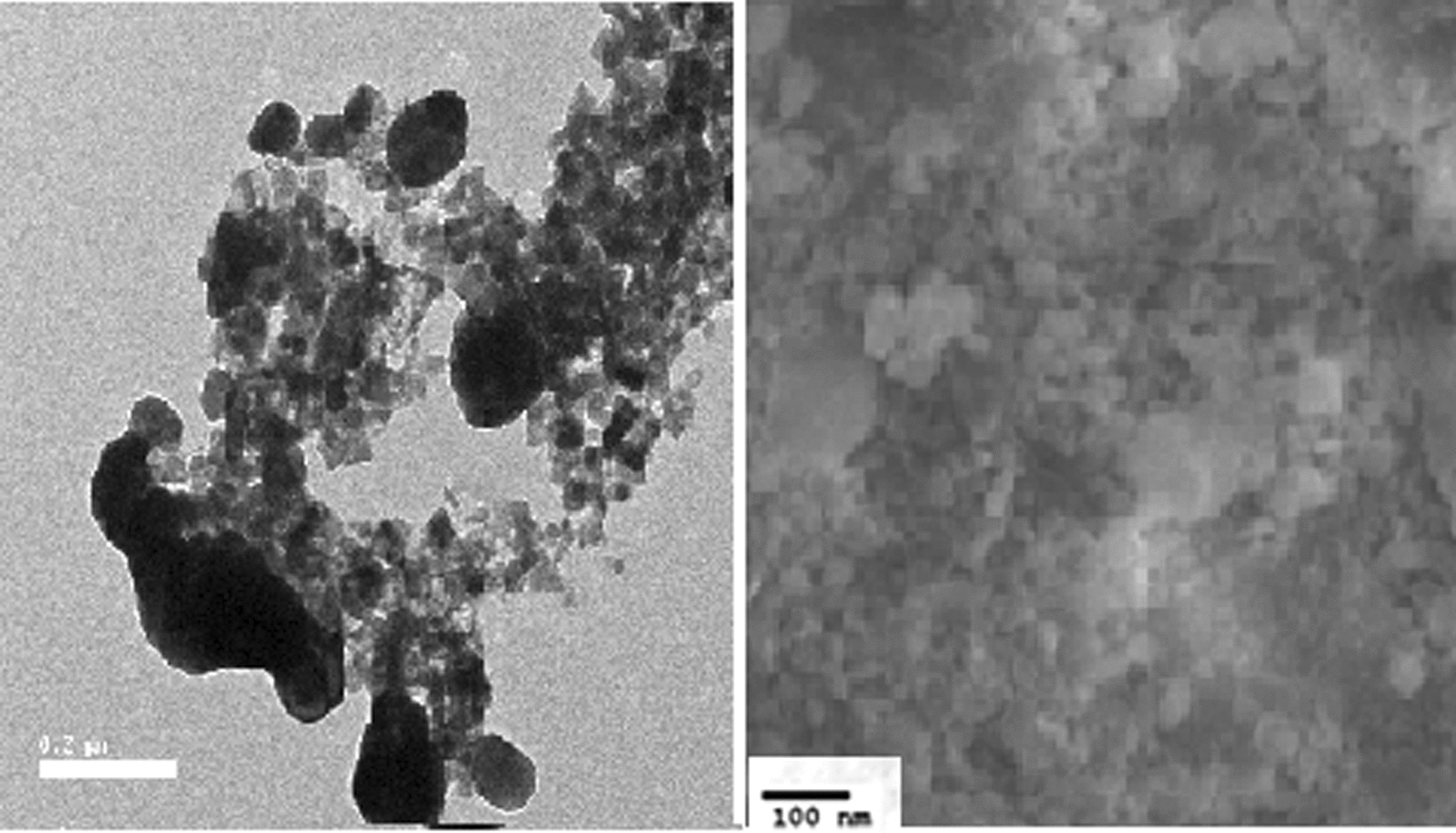
Scanning electron microscope examination for the resultant bionanosilver particles produced by the filtrate of the *Lactobacillus acidophilus*

The size of the biosynthesized AgNPs ranged from 33 to 90 nm, according to SEM observations (Fig. 2).

### XRD and FTIR analysis

**Fig. 3:**
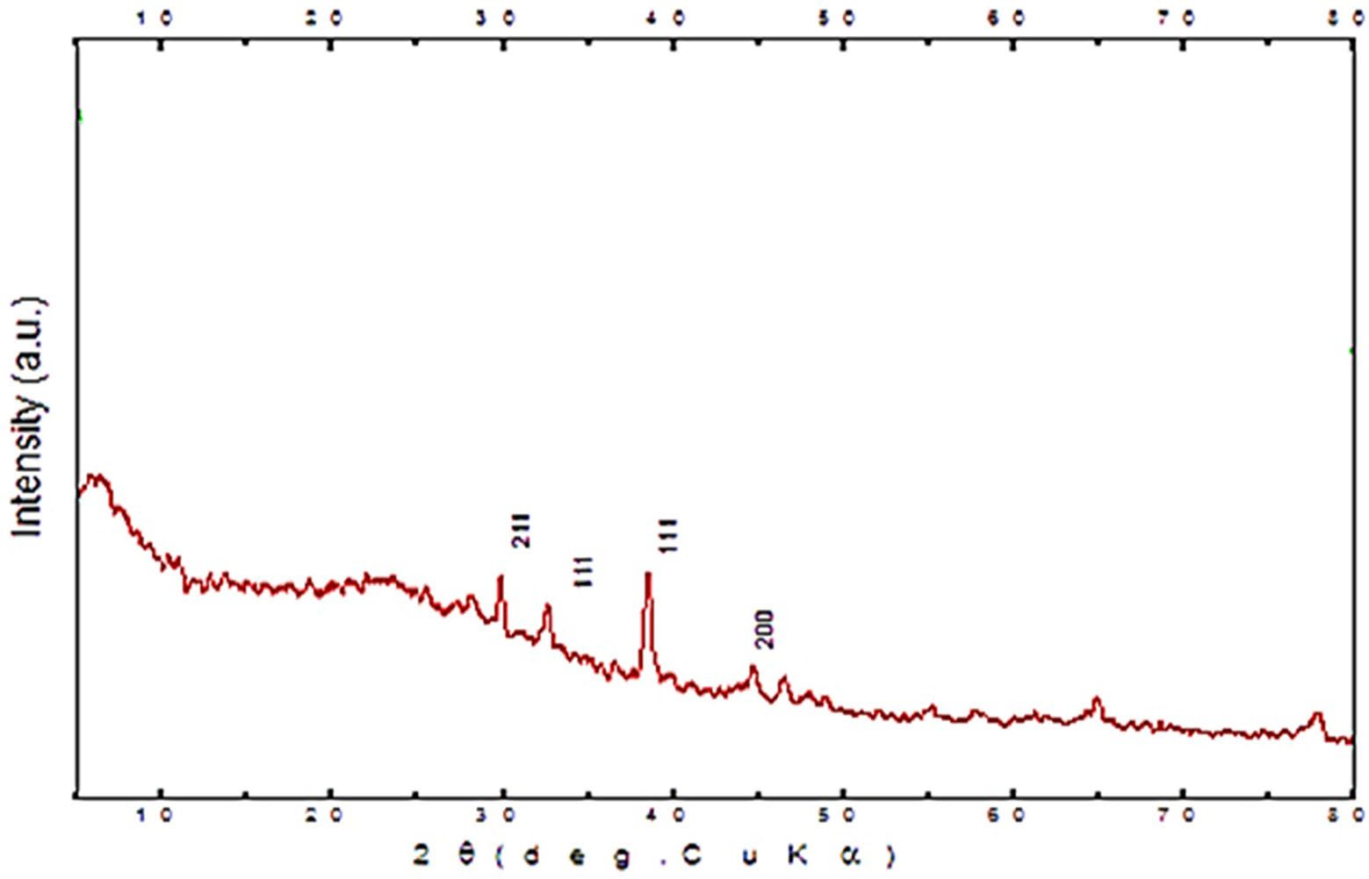
XRD patterns of nanoparticles prepared using *Lactobacillus acidophilus*

**Fig. 4:**
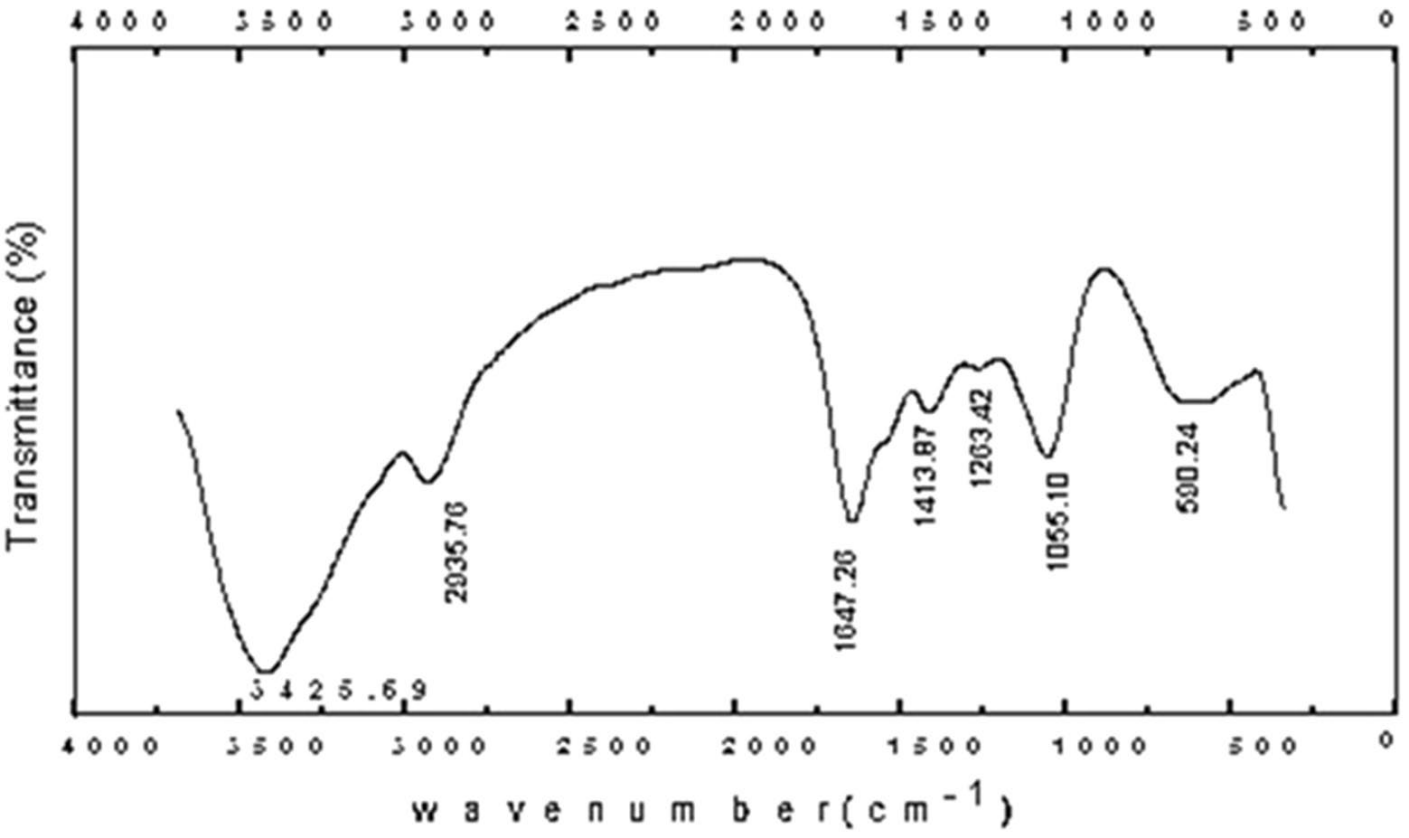
FTIR spectra of nanoparticles prepared using *Lactobacillus acidophilus*

The crystalline nature of biosynthesized silver NPs was confirmed by XRD examination using *L. acidophilus* showing diffraction peaks at 2θ values at = 29.8 °C, 32.3 °C, 38.9 °C, and 44.7 °C. Silver crystal planes, respectively which are indexed as crystalline silver (Fig. 3), also Fig. 4 showed the FTIR spectra of biosynthesized silver nanoparticles. The number of functional biological groups responsible for the reduction of Ag ions, which act as capping or stabilizing agents, was discovered through spectral analysis utilizing FTIR. Absorbance bands of *L. acidophilus* with Ag ions observed at 3425.60/cm were assigned to O–H (s) groups, 1647.26/cm assigned to carboxylic group (OHC=O), and 2935.75/cm assigned to alkyl group (–CH3). These peaks in the FTIR spectra show that *L. acidophilus* molecules with OH and CO groups play an important role in the reduction and stability of NPs.

### Cytoxicological impacts of synthesized bionanosilver

When analyzing several physiological indicators, the *L. acidophilus* nanoparticles had little harmful effect on the treated rats when compared to the control non-treated group. The parameters investigated were ALT, AST, creatinine, albumin, and urea (Table 1) as followed:

**Table 1:** Physiological parameters of the animals treated with generated AgNPs

Group	Mode of induction	Weight (g)	Albumin (g/dL)	ALT (U/L)	AST (U/L)	Creatinine (mg/dL)	Urea (mg/dL)
Control group	Control after 1 week	200	0.95 ± 0.015	27.1 ± 0.02	115.74 ± 0.015	0.38 ± 0.01	18.24 ± 0.015
	Control after 2 weeks		0.95 ± 0.02	27.13 ± 0.01	115.76 ± 0.01	0.367 ± 0.015	18.21 ± 0.015
Group 1	Intragastric 200 mg/kg AgNPs 3 times for 1 week	200	0.38 ± 0.015*	48.65 ± 0.01*	235.14 ± 0.025*	1.17 ± 0.021*	38.29 ± 0.0.015*
Group 2	Intragastric 200 mg/kg AgNPs 3 times for 2 weeks	200	0.17 ± 0.01*	71.21 ± 0.0153*	324.62 ± 0.015*	1.65 ± 0.01*	53.9 ± 0.01*

* indicates statistically significant variations at *P* < 0.05

### Impacts of AgNPs administration on AST and ALT

The impact of utilization of AgNPs on levels of AST and ALT, respectively declared that there was a significant increase of serum AST level in 200 mg/kg AgNPs groups treated for 14 days (324.62 ± 0.015) and 7 days (235.14 ± 0.025), respectively, compared to the control groups (115.76 ± 0.01) and (115.74 ± 0.015) at 14 and 7 days, respectively, also there was a significant increase of serum ALT level in 200 mg/kg AgNPs groups treated for 14 days (71.21 ± 0.0153) and 7 days (48.65 ± 0.01), respectively, compared to the control group (27.1 ± 0.02) and (27.13 ± 0.01) at 7 and 14 days respectively.

### Impacts of AgNPs administration on serum albumin

There was a considerable drop in albumin levels throughout the group treated with 200 mg/kg AgNPs for 7 days (0.38 ± 0.015) and 14 days (0.17 ± 0.01), respectively, compared to the control groups (0.95 ± 0.015) and (0.95 ± 0.02) at 7 and 14 days respectively.

### Impacts of AgNPs administration on serum creatinine and urea

Serum levels of creatinine and urea were showed a significant increase in 200 mg/kg AgNPs treated rats. Creatinine levels in the blood increased significantly in groups treated for 14 days (1.65 ± 0.01) and 7 days (1.17 ± 0.021), respectively, compared to the control group (0.38 ± 0.01) and (0.367 ± 0.015) at 7 and 14 days, respectively. Serum urea levels showed a significant increase in treated animals (53.9 ± 0.01) and (38.29 ± 0.015) after 14 and 7 days, respectively, as compared to control non-treated groups at 7 days (18.24 ± 0.015) and 7 days (18.21 ± 0.015).

### Antibacterial activity of bionanosilver against four pathogenic bacterial strains compared with three commercial antibiotics

As shown in Table 2, the AgNPs formed with the filtrate of the *L. acidophilus* showed high activity against the four MDR bacteria when compared with the chemically synthesized antibiotics. The concentration of 25% showed high activity against the four pathogenic bacteria, and this activity was increased with the concentration of Ag used. The lowest inhibition zones obtained by the AgNPs were 5 mm (25 µg/mL) and the highest activity was obtained with 100% concentration (20 mm); in contrast the lowest activity obtained by the chemical antibiotics was 4 mm (200 µg/mL) and the highest activity was 10 mm (streptomycin). Collectively, the activity of the biosynthesized nanosilver was 200% greater as compared with the chemical antibiotics, and the four examined bacterial strains showed sensitivity with different manners.

**Table 2:** Comparison between the impact of AgNPs and antibiotics on tested bacteria

The average size of the growth inhibitory zone (mm)				
	*Proteus vulgaris*	*Escherichia coli*	*Staphylococcus aureus*	*Klebsiella pneumonia*
Biogenic antibiotics (200 µg/mL)				
Ampicillin	5	0	5	0
Gentamycin	8	7	4	0
Streptomycin	10	9	9	0
Nanosilver concentration (µg/mL)				
10	5	0	0	5
25	5	6	7	6
50	11	13	12	11
100	19	19	18	20

### DD-PCR

**Fig. 5:**
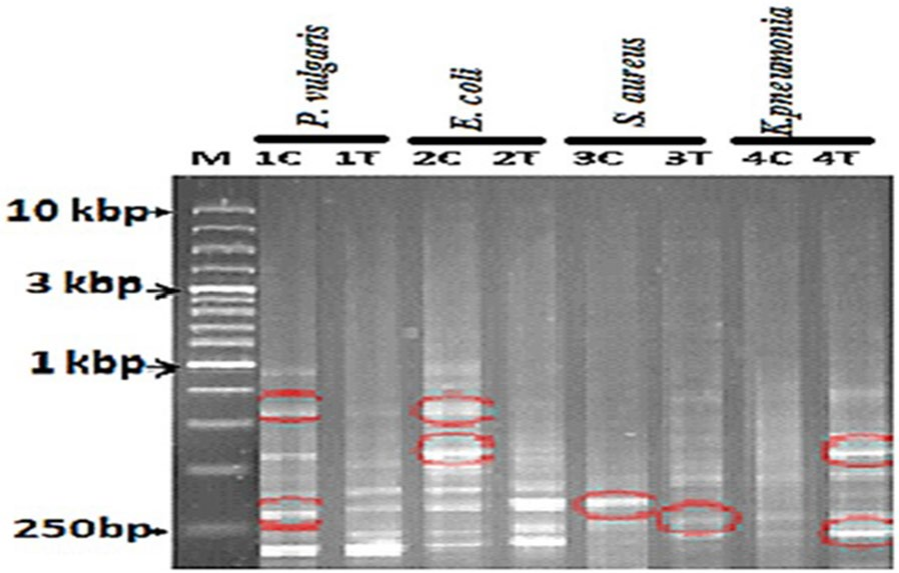
Differential Display band pattern for the treated and non-treated bacterial cells with the biosynthesized nanosilver. M (10Kbp DNA marker), 1: *P. vulgaris*, 2: *E. coli*, 3: *S. aureus*, 4: *K. pneumonia*. C: means control (or non-treated), T: treated with nanosilver

Data presented in Fig. 5 show that treated and non-treated bacteria have different DNA patterns. Primers *mec*A and beta-lactam genes scan the transcriptome of the treated cells when compared with non-treated ones. Several genes were found to be down-regulated, whereas others were activated at the same time. The most interesting thing is that all the amplified bands in the same bacterial species are polymorphic and there are no monomorphic bands. The species which are characterized by shutdown of the most amplicons are *E. coli* followed by *P. vulgari*s and *S. aureus*, but in case of *K. pneumoniae*, at least three genes are induced. The induced genes could be transcription factors such as zinc finger protein but the genes which are completely suppressed are mostly defense or DNA resistance genes such as *mec*A gene.

### Up- and down-regulated genes

Impact of AgNPs on the transcriptomes in the treated bacteria compared to the untreated bacteria was clear, and it was based the suppressed and activated genes. In the case of *P. vulgari*s, two genes were suppressed, one approximately 820 bp and the second one about 270 bp. In *E. coli*, two genes were completely suppressed, one 820 bp (the same molecular size of that *P. vulgaris*) and the second one 540 bp. Accordingly, *S. aureu*s had a gene about 300 bp that was completely suppressed and another gene about 250 bp was induced. Finally, in the case of *K. pneumonia*e, two genes were up-regulated (induced), one 540 bp and the second one 200 bp.

### Sequencing and sequence analysis

**Fig. 6:**
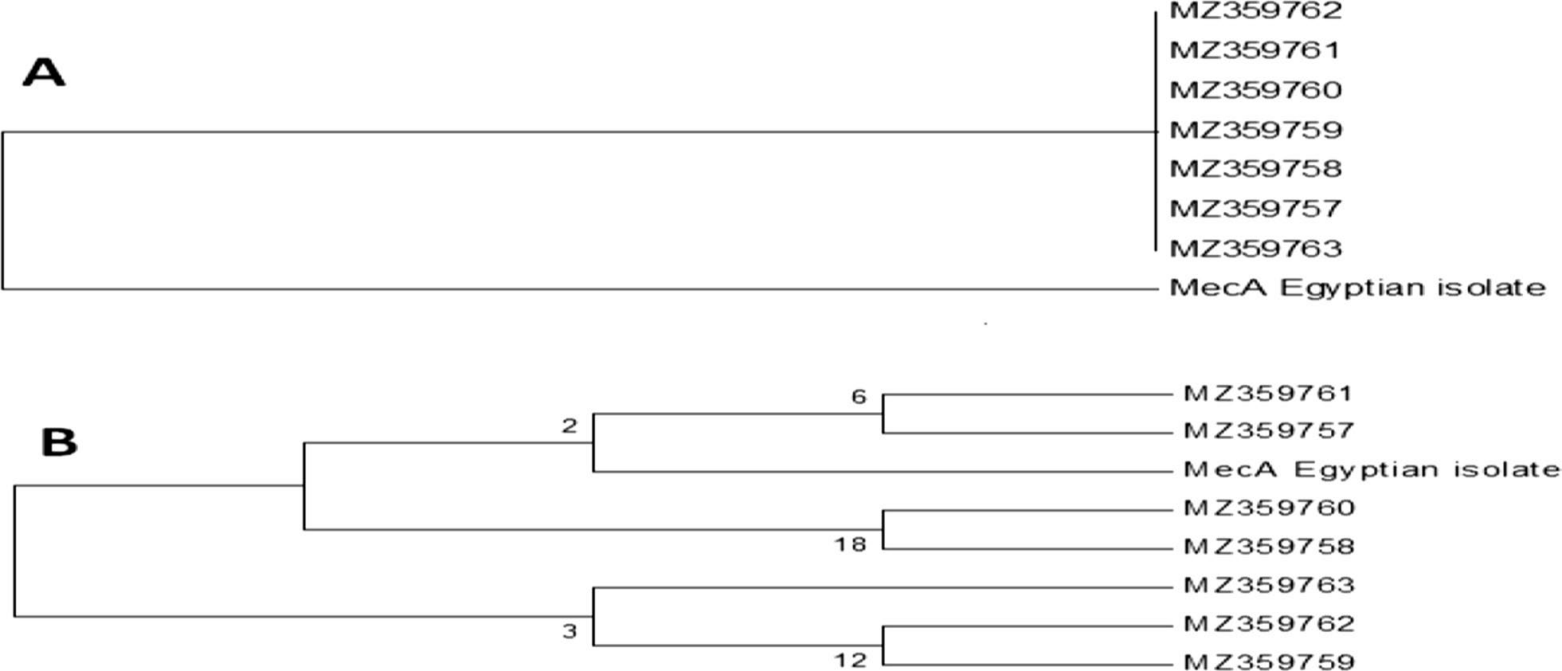
Phylogentic tree for the isolated down regulated *mec*A gene from *S. aureus* compared with other *meg*A genes listed on Gene Bank. **A** Phylogeny constructed based on the DNA nucleotide sequence. **B** Phylogeny constructed based on the deduced amino acids of the obtained DNA sequence

**Fig. 7:**
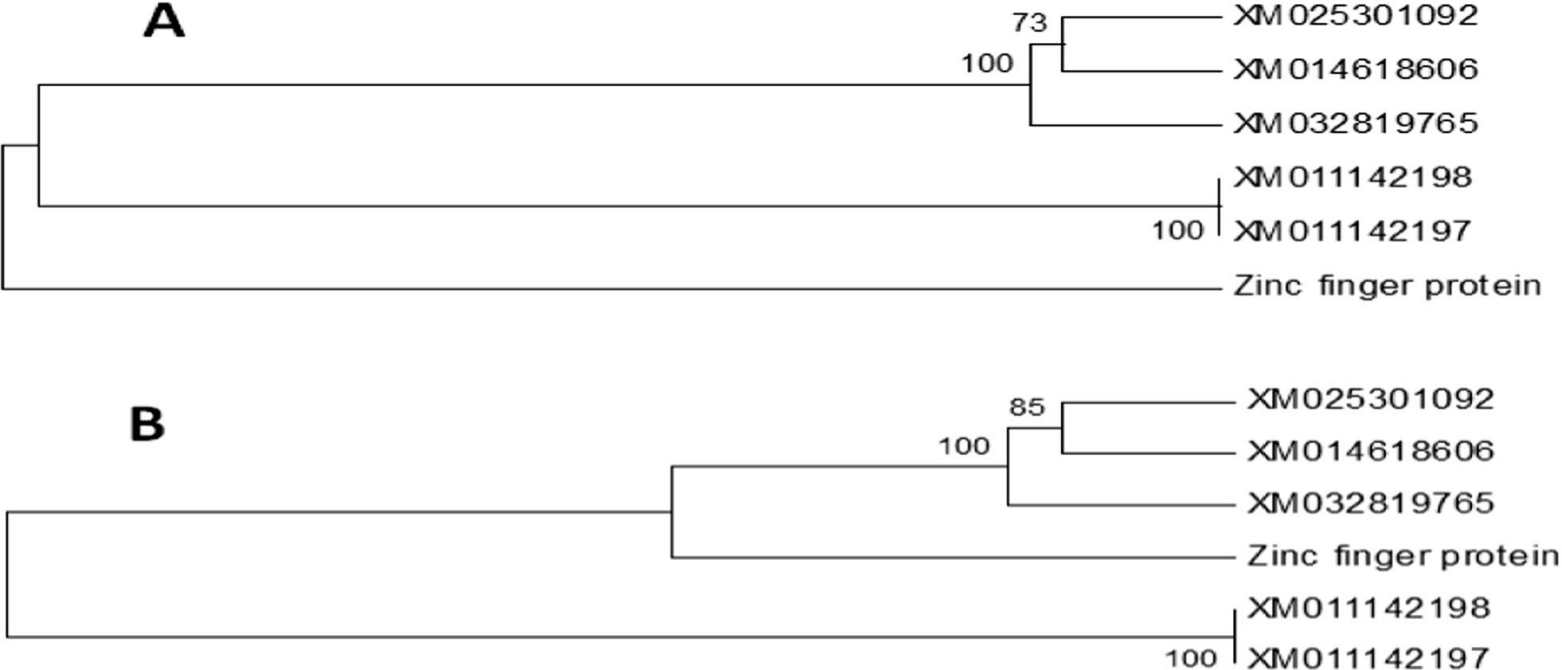
Phylogentic tree for the isolated upregulated Zinc finger protein gene isolated from *K. pneumonia* compared with other Zinc finger protein genes listed on Gene Bank. **A** Phylogeny constructed based on the DNA nucleotide sequence. **B** Phylogeny constructed based on the deduced amino acids of the obtained DNA sequence

The DNA sequence of the down-regulated gene isolated from control *S. aureus*, GenBank MZ748472-MZ748473, was identified as *mec*A. When the sequence of the *mec*A gene of the *S. aureus* (Egyptian isolate) was compared with other *mec*A genes listed in GenBank (Fig. 6), it was observed that gene is closely related to the gene *S. aureus* MZ2359758 isolate (Baghdad, Iraq). In the case of the zinc finger protein (Fig. 7), it is clear the genes isolated from *Odontomachus brunneus* in Florida (USA) and the Egyptian one are highly similar.

## Discussion

Silver nitrate reduction and nanosilver formation was in accordance with Abdel-Azeem et al. (2020) who proved that the color shift in the reaction mixture confirmed the production of the AgNPs. And size of the biosynthesized AgNPs which ranged from 33 to 90 nm by SEM was nearly in the same rang obtained by El-Aassar (2013), who found that the prepared AgNPs size ranged between 50 and 80 nm while higher than what El-Deeb et al. (2015) found when they produced AgNPs with sizes ranging from 20 to 60 nm and may be attributed to the method and source of synthesis of AgNPs. About XRD and FTIR analysis of biosynthesized silver NPs was an accurate method for characterization of AgNPs as reported by Abdel-Azeem (2020) also agreed with the findings of El-Sherbiny et al. (2016) and Yusof et al. (2020), who used FTIR to characterize synthesized AgNPs.

*Lactobacillus acidophilus* nanoparticles had little harmful effect on the treated rats when compared to the control non-treated group. The physiological parameters investigated were ALT, AST, creatinine, albumin, and urea (Table 1) as well as Fig. 5A–E, this was in accordance with Lee et al. (2010) who reported that AgNPs exposure changed the expression of many genes linked to motor neuron problems, neurodegenerative illness, and immune cell function, indicating possible neurotoxicity and immunotoxicity from contact with AgNPs. However, Stebounova et al. (2011) reported that after 10 days of AgNP treatment, mice showed no signs of lung inflammation or cytotoxicity. This may be attributed to the nature and source for biosynthesis of AgNPs.

Antibacterial activity of bionanosilver against four pathogenic bacterial strains compared with three commercial antibiotics was obvious as shown in Table 2, when Manzoor et al. (2016) treated *E. coli* cells with ZnO, they got the same findings. AgNPs are powerful antimicrobials that kill a wide range of Gram-negative and Gram-positive MDR bacteria (Burrell et al. 1999; Yin et al. 1999) (Percival et al. 2007). Li et al. (2009) showed that AgNPs are being investigated as a valuable material because of their potent antibacterial properties. To study the antibacterial activity of Ag/porous poly(l-lactic acid fibrous membranes, antibacterial tests were performed using *E. coli* and *Staphylococcus*, two bacteria that commonly invade wounds and other tissues, and good results were obtained (Yeo et al. 2003). This agrees with studies published showing that AgNPs bind to the negatively charged bacterial cell wall, rupturing it, and causing cell death via denaturation of cell protein (Lin et al. 1998, Zawrah et al. 2011). According to recent findings, silver nanoparticles interact with bacterial cells in a shape-dependent manner (Pal et al. 2007).

As shown in Fig. 5, treated and non-treated bacteria have different DNA patterns, Pillai et al. (2014) investigated the effects of nanosilver on the transcriptome, proteome, and phenotypic of *Chlamydomonas reinhardtii*. They found that microalgae cells treated with nanosilver responded positively, as evidenced by their transcriptome and proteome. Both the genome-wide transcriptome and proteome of microorganisms treated with nanosilver changed, according to Ankley et al. (2010), and these alterations should aid in determining the molecular pathways resulting from nanosilver stress. Furthermore, nanosilver has been shown to suppress RNA polymerase activity and RNA transcription in treated cells (Wang et al. 2013).

Interestingly, similarity in the isolated genes and the others listed in GenBank was obtained when their amino acids were compared. These results indicate that these genes may be different in nucleotide structure but similar in amino acid structure. It is well known the *mec*A is one of the DNA resistance genes that enable *S. aureus* to resist the many antibiotics. Similarly, the zinc finger protein is considered as the most common transcription factor in bacterial lineages, and it has a multiple functions as virulence, symbiosis, and/or cell cycle transcription. So AgNPs biosynthesized with *L. acidophilus* are environmentally friendly, fast-acting, and economical. They have antibacterial properties against some MDR Gram-positive and Gram-negative pathogenic microorganisms and may become more widely used in treatment and control of serious diseases caused by them.

## Data Availability

All data generated or analyzed during this study are included in the submitted manuscript.
